# Characteristic Assessment of Diesel-degrading Bacteria Immobilized on Natural Organic Carriers in Marine Environment: the Degradation Activity and Nutrient

**DOI:** 10.1038/s41598-017-08832-y

**Published:** 2017-08-17

**Authors:** Jianliang Xue, Yanan Wu, Zhixiu Liu, Menglu Li, Xiyu Sun, Huajun Wang, Bing Liu

**Affiliations:** 1 College of Chemical and Environmental Engineering, Shandong University of Science and Technology, Qingdao, Shandong P.R. China; 2 College of Mechanical and Electronic Engineering, Shandong University of Science and Technology, Qingdao, Shandong P.R. China

## Abstract

Oil spill has led to severe environmental and ecological problems. Due to the harsh environmental conditions, the bioremediation technology is not successfully used to remedy the oil spill in marine environment. In this study, immobilization technology was used to immobilize bacteria on natural organic carriers (i.e., wood chips and maize straw). The higher surface area of in wood chips leads to larger biomass density (0.0242 gVSS/g) than that of maize straw of 0.0097 gVSS/g carrier. Compared with biodegradation efficiency of free bacteria (44.79%), the immobilized bacteria on wood chips and maize straw reached to 73.39% and 52.28%, respectively. The high biological activity of the immobilized bacteria can be also explained by nutrients, such as TN (total nitrogen) and TP (total phosphorus), released from wood chips and maize straw, which was 8.83 mg/g and 5.53 mg/g, 0.0624 mg/g and 0.0099 mg/g, respectively.

## Introduction

The discharge of oily wastewater or spill from exploitation and/or transportation inevitably threatens environmental and ecological safety. Thus, many technologies have been developed to resolve this severe problem. At present, many types of hydrocarbon-degrading bacteria that can utilise different types of hydrocarbons as carbon and energy sources in metabolism exist in the marine environment^1, 2^. Thus, among the different remediation technologies (e.g. bioremediation, physical remediation and chemical remediation), bioremediation is proposed as an environmentally friendly and high-process-efficiency strategy for remedying the oil spill in a simulated marine environment. However, the harsh environmental conditions in the marine environment (e.g. wave and tidal movements) are unsuitable for the enrichment of hydrocarbon-degrading bacteria. In addition, except for carbon source, nutrients are necessary for hydrocarbon-degrading bacteria. Several researchers showed that the degradation of petroleum hydrocarbons is the highest when both sources are added (C/N/P = 150:6:1)^3^. In fact, the lack of nutrients (e.g. nitrogen and phosphorus) limits the physical activity of hydrocarbon-degrading bacteria in the marine environment^4^. Thus, bioremediation has not been successfully utilised in real oil-polluted seawater^5, 6^.

According to the previous literature^7–10^, immobilization is the optimal bioremediation technology because of its many advantages, such as high biomass density, enhanced microbial stability and easy separation from the reaction environment. Thus, immobilization technology has potential prospects for the biological treatment of various pollutants^11^. Most reports indicated that immobilization can be achieved in petroleum-contaminated soil bioremediation and other bioremediation fields^12, 13^. Several studies reported about implementing immobilization to degrade oil in the marine environment. Liu *et al*. applied immobilized (dominant CC-HCCH11) bacteria to treat petroleum hydrocarbon–diesel in baffled flask tests, with the highest degradation percentage of 78%^6^. Wang *et al*. reported that the immobilized bacteria exhibit good salinity tolerance, with a diesel oil removal efficiency exceeding 85%^14^. At present, considerable attention has been focused on developing immobilization carriers with several specific properties, including strength, chemical and thermal stability, durability and surface properties. However, the lack of nutrients (e.g. nitrogen and phosphorus) limits the activity of immobilized bacteria in the treatment of oil pollution. Chen (2012) applied polyurethane–polyurea copolymer as an immobilization carrier and determined that the degradation efficiency is only 47.25% without providing sufficient nutrients^15^.

In the present study, different natural organic carriers were used to immobilize diesel-degrading bacteria and degrade diesel in seawater effectively. Simultaneously, the nutrients released from natural organic carriers during biodegradation were investigated, and the relationship between nutrient and bioactivity was analysed. This paper reports a new pathway of achieving high bioactivity and obtaining nutrients by using natural organic carriers; thus, a useful strategy to remedy oil-polluted seawater is provided.

## Materials and Methods

### Enrichment of diesel-degrading bacteria

Seawater samples of isolated diesel-degrading bacteria were collected from Tang Island Bay, Qingdao, Shandong Province, China. Firstly, mineral salt medium (MSM) containing 0.6 g/L Na_2_HPO_4_, 0.2 g/L KH_2_PO_4_, 4.0 g/L NaNO_3_, 1.0 g/L, yeast powder and trace element solution was prepared. The trace element solution consisted of 1 mL 1.11 g/L CaCl_2_, 1 mL 1.52 g/L FeSO_4_ and 2 mL 3.60 g/L MgSO_4_. Then, diesel-degrading bacteria were enriched by adding the 5 mL of seawater sample to a 100 mL Erlenmeyer flask with 100 mL of sterile MSM and 1 mL of diesel oil. The Erlenmeyer flask was placed in a shaker (310 K, 160 rpm) for 7 days. Afterward, a 10 mL aliquot of the incubated sample was extracted from the medium and then diverted to the fresh liquid mineral medium. Five subsequent enrichments were conducted to isolate diesel-degrading bacteria selectively. The adequate number of diesel-degrading bacteria was confirmed by the dilution plate method. In the experiment, the number of bacteria was approximately 8–9 × 10^8^ CFU/mL.

### Experimental design

In this work, three experimental designs were applied to assess the superiority of natural organic carriers and to analyse the biodegradation mechanism in seawater. In consideration of their nutrients and surface properties, wood chips and maize straw were selected as natural organic carriers in this study.

#### Design I

Biodegradation of bacteria immobilized on wood chip carriers

Impurities (e.g. some organic matter) could be adsorbed on wood chips. Firstly, the wood chips were broken into pieces (approximately 100–400 µm) and washed sequentially with ethanol and distilled water several times to prevent the impurities from affecting the growth and immobilization of the bacteria. The wood chips were dried at 378 K for 2 h. Secondly, 0.25 g of wood chips were placed in a 250 mL flask containing 100 mL of immobilized culture medium. The immobilized culture medium used in this study consisted of 10.0 g/L peptone, 4.0 g/L beef paste and 5.0 g/L NaCl. This medium is a widely used natural medium, in which beef paste is the source of carbon, phosphate and vitamins; peptone is the source of nitrogen and vitamins; and NaCl is the source of inorganic salt. Afterwards, the flask with the wood chips and medium was sterilised in an autoclave (394 K, 20 min). Thirdly, after cooling to ambient temperature, some hydrocarbon-degrading bacteria were inoculated. The flask with the wood chips and the bacteria was cultivated at 303 K and 160 rpm until a biofilm formed on the carrier. The wood chips were washed with saline solution by centrifugation at 4000 rpm for 10 min to remove microorganisms that were not immobilized on the chip. The microorganisms immobilized on the wood chips were harvested by centrifugation at 8000 rpm for 10 min. The number of bacteria was approximately 8 × 10^8^ CFU/mL in the experiment as determined using the dilution plate method. Fourthly, the carrier immobilizing diesel-degrading bacteria was washed and centrifuged twice with normal saline at 4000 rpm. Approximately 25 same samples were prepared using the previously mentioned steps.

Then, a certain number of bacteria were immobilized on the wood chips because of the vesicular structure of the wood chips. These diesel-degrading bacteria in the carriers were placed in 100 mL flasks containing 100 mL of sterilised seawater. Then, 1 mL of diesel was synchronously injected. Afterwards, these flasks were placed in a shaker at 310 K and 120 rpm, and the values of the indexes were analysed at different times. Simultaneously, the same amount of wood chips without inoculation was run, and the diesel loss of abiotic factors was observed. Finally, in sequence, a stack of five flasks was taken out from the incubator after 5, 7, 10, 15 and 20 days. The determined indexes were subtracted from the data of the abiotic control experiment to obtain the true values of the indexes further (including the degradation efficiency of diesel, enzyme activity, total nitrogen [TN] and total phosphorus [TP]).

#### Design II

Biodegradation of bacteria immobilized on maize straw carriers

The biodegradation experiment of bacteria immobilized on maize carriers followed the previously mentioned steps.

#### Design III

Biodegradation of free bacteria

The number of free bacteria was determined and compared with that of immobilized bacteria in this study (approximately 0.25 g/1 mL). In contrast to the biodegradation of bacteria immobilized on natural organic carriers, free bacteria were directly injected into 100 mL flasks containing 100 mL of sterilised seawater. Then, 1 mL of diesel was synchronously injected. Afterwards, these flasks were placed in a shaker at 310 K and 120 rpm, and the values of the indexes were analysed at different times. Simultaneously, the same amount of wood chips without inoculation was run, and the diesel loss of abiotic factors was observed. Finally, in sequence, a stack of five flasks was taken out from the incubator after 5, 7, 10, 15 and 20 days. The indexes (including the degradation efficiency of diesel, enzyme activity, TN and TP) were determined. The measured data were subtracted from the data of the abiotic control experiment to obtain the true values of the indexes further.

### Analysis method

#### Degradation efficiency of diesel

Diesel was the main subject of this investigation, and the methods used to analyse diesel in the samples were as follows:

Firstly, the carriers taken out from flask were washed many times with 10 mL of *n*-hexane. Then, the washing liquid was poured into the flask again and shaken for 10 min. Afterwards, the liquid sample was poured into a separating funnel. Finally, the extraction liquid was obtained and diluted 100 times. The concentration of diesel was determined by measuring absorbency at 250 nm via ultraviolet spectrophotometry. The concentration of diesel was calculated on the basis of the absorbency values^16^. According to the equation, the degradation efficiency of diesel was calculated as follows:

1 $$Y=\frac{{C}_{0}-{C}_{1}}{{C}_{0}}\times 100 \% $$

where *Y* is the degradation efficiency of diesel (%), *C*_0_ is the initial concentration of diesel (mL/L) and *C*_1_ is the final concentration of diesel (mL/L).

#### Biomass densities of the carriers

Biomass density was estimated and/or measured as previously described^17^. The biomass density of the carrier samples was determined on the basis of the volatile suspended solids (VSS) per carrier. VSS is the organic matter content of the total suspended substance, and VSS per carrier is the VSS per gram carrier.

#### Specific surface area and pore size distribution

Firstly, the carrier was dried in liquid nitrogen environment. Then, the surface properties (including specific surface area and median diameter of the adsorption pore) were measured with a surface aperture analyser ASAP2020M.

#### Determination of dehydrogenase activity

The extracts were harvested by centrifugation at 8000 rpm for 10 min, washed twice and then re-suspended in 0.85% NaCl to obtain the suspension. Firstly, 2 mL of the supernatant was removed and then placed in a test tube. Afterwards, 2 mL Tris–HCl buffer solution, 2 mL 0.1 M glucose solution and 2 mL 0.5% TTC were sequentially added to the test tube and then incubated (310 ± 1 K, 5 h). Secondly, the test tube was removed and added with a small amount of concentrated sulphuric acid to stop the reaction. Thirdly, the test tube was added with 5 mL of ethyl acetate and then shaken for a few minutes. Finally, the organic solution of the upper layer was removed for determination. The dehydrogenase activity concentration was determined by ultraviolet spectrophotometry^18^.

#### Determination methods of nitrogen and phosphorus

After pouring out the diesel in the flask, the residual liquid was pumped through vacuum extraction filtering, and the 0.22 µm filter membrane was adopted. Part of the extraction solution was selected, and the concentration of TN was determined. Firstly, some of the extracts were placed in a 25 mL colorimetric tube. Secondly, 5 mL of alkaline potassium (40.0 g of potassium persulphate, 15.0 g of NaOH and 1000 mL of H_2_O) was added to the colorimetric tube, which was placed in the autoclave (394 K, 30 min). Thirdly, 1 mL of 1.2 M hydrochloric acid solution was added to the colorimetric tube and then diluted. Fourthly, the concentration of TN was determined by measuring the absorbency value at 220 and 275 nm via UV spectrophotometry. Finally, the concentration of TN was calculated on the basis of the absorbency value.

Part of the extraction solution was selected, and the concentration of TP was determined. Firstly, some of the extracts were placed in a 50 mL colorimetric tube. Secondly, 4 mL of 50 g/L potassium persulphate solution was added to the colorimetric tube, which was placed in the autoclave (394 K, 30 min). Thirdly, 1 mL of 100 g/L ascorbic acid solution and 2 mL of ammonium molybdate solution (13.0 g of ammonium molybdate, 0.35 g of antimony potassium tartrate, 200 mL of H_2_O and 300 mL of 9.2 M sulphuric acid) were added to the colorimetric tube. Fourthly, the colorimetric tube was cooled to room temperature for 15 min. The concentration of phosphorus was determined by measuring the absorbency value at 700 nm through the spectrophotometric method. Finally, the concentration of TP was calculated on the basis of the absorbency value.

## Result

### Surface properties of the carriers and immobilization of bacteria

**Figure 1:**
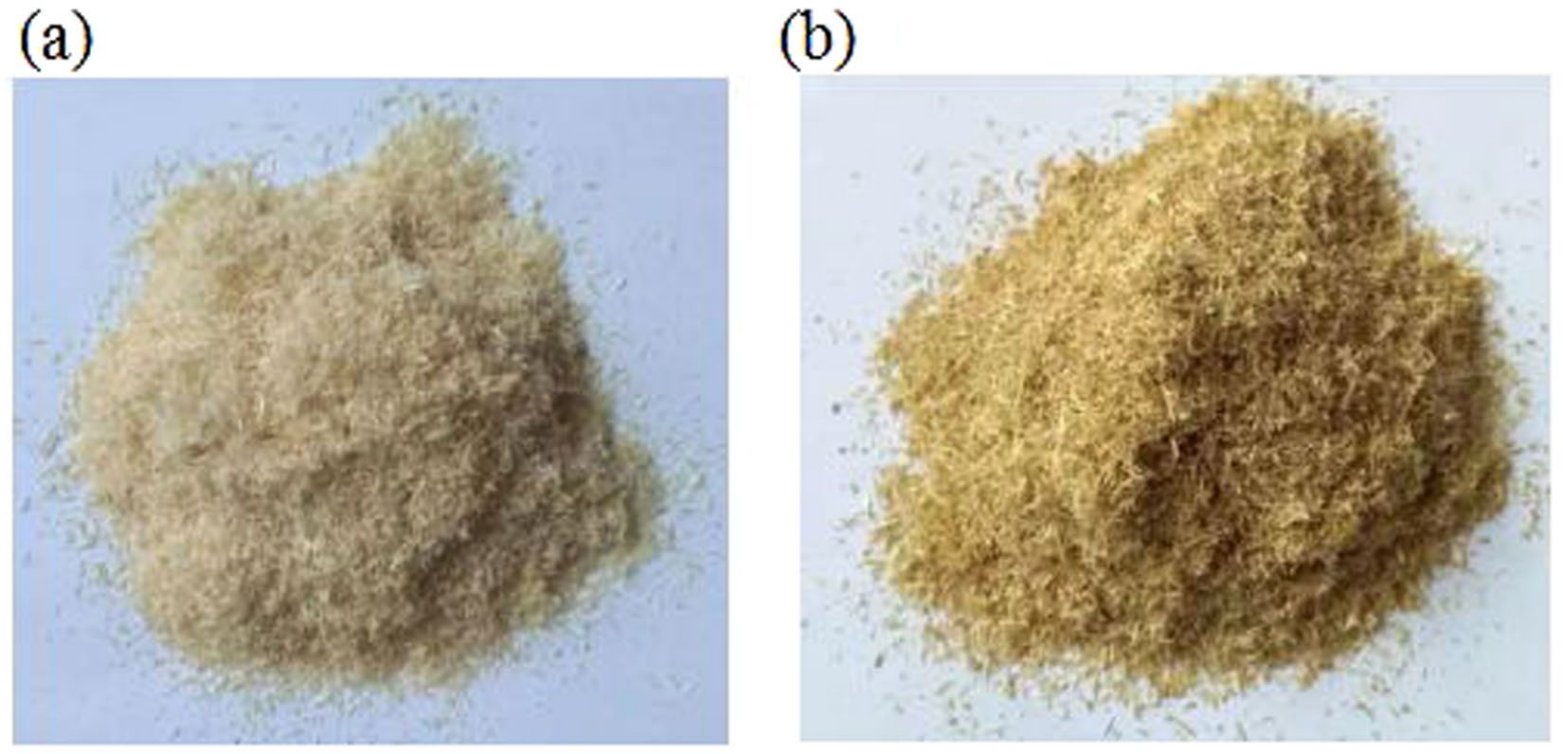
Photographs of different carriers. (**a**) Wood chips; (**b**) Maize straw.

**Figure 2:**
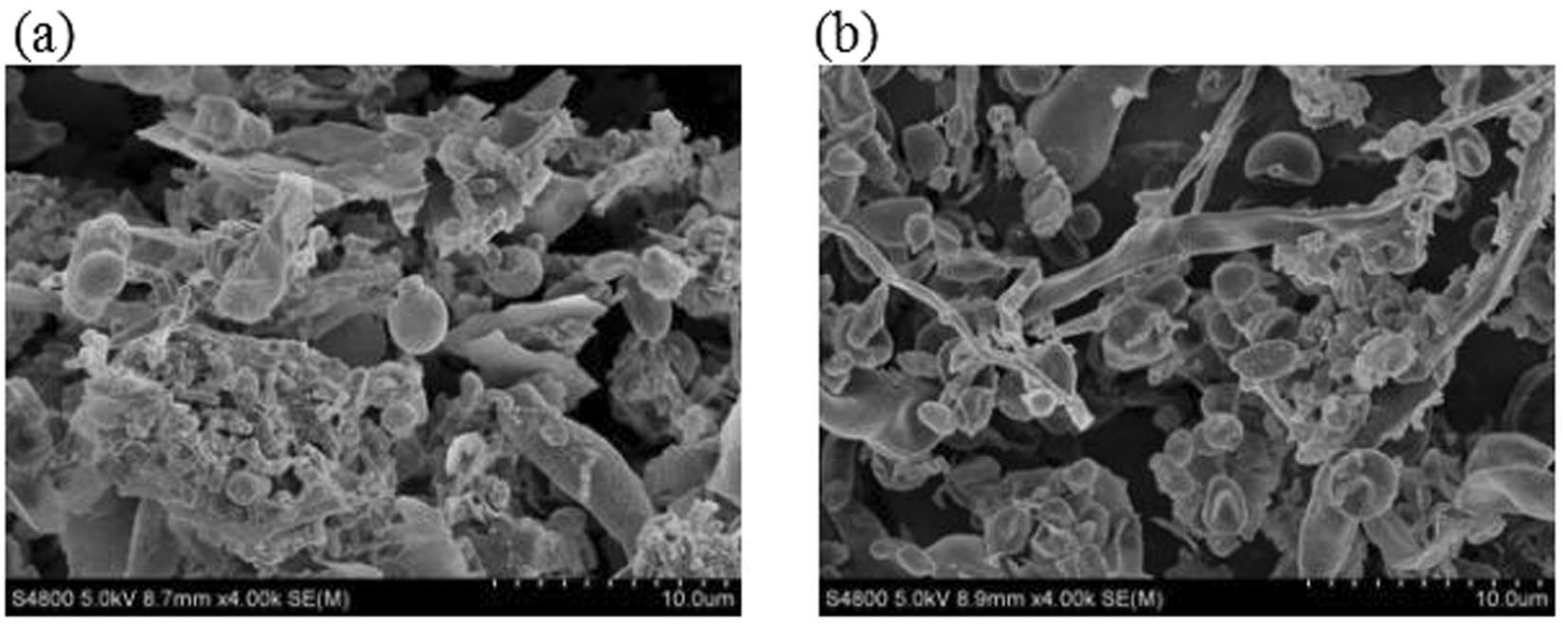
SEM photographs of different carriers. (**a**) Wood chips; (**b**) Maize straw.

The surface properties of the wood chips and maize straw showed that these carriers were suitable for immobilizing bacteria (Table 1, Figs 1 and 2). The surface properties of these carriers (e.g. specific surface area and median diameter of the adsorption pore) and the amount of biomass present in the carriers were estimated, as shown in Table 1. As shown in Table 1, the wood chips and maize straw contributed to favourable adsorption properties. The biomass densities of the wood chips and maize straw were 0.0242 and 0.0097 g VSS/g carrier, respectively. In addition, the specific surface area of the wood chips was greater than that of the maize straw and the median diameter of the adsorption pore of the wood chips was larger than that of the maize straw. The surface properties contributed favourably to achieve a high biomass density. According to the biomass density of the wood chips and maize straw, wood chips are effective as immobilization carriers.

**Table 1:** Surface properties of the carriers and Biomass densities.

Carrier		Surface properties of the carriers		Biomass density (gVSS/gcarrier)
	medium diameter/μm	specific surface area (m^2^/g)	medium diameter of adsorption pore (nm)	
Wood chips	10–40	0.4625 ± 0.0327	8.16	0.0242 ± 0.00127
maize straw	10–40	0.4164 ± 0.0219	7.89	0.0097 ± 0.0007

In addition, the median diameter of the adsorption pore was only 7.89–8.16 nm, which showed that the bacteria were initially absorbed on the surface, gradually bred and then formed a biofilm. Finally, the SEM images showed that the biofilm was immobilized on the carrier (Fig. 2). Immobilised bacteria can be adsorbed on the carrier. As shown in Fig. 2, the bacteria grew in lumps. The morphology of the microorganisms was homogeneous, and their surface was smooth. The activity of the microorganisms was unaffected.

### Biodegradation of total petroleum hydrocarbon–diesel by immobilized and free bacteria

**Figure 3:**
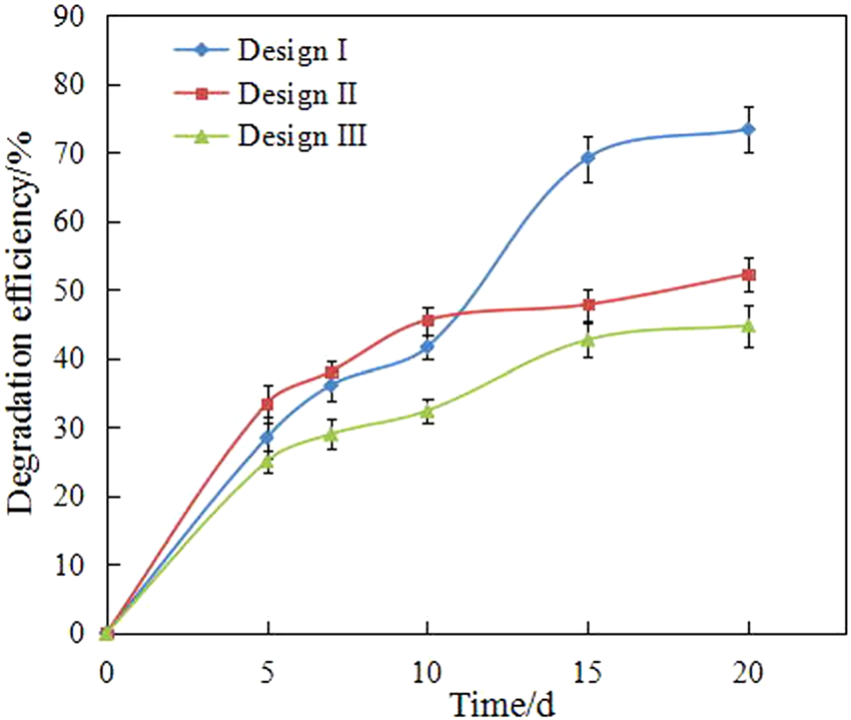
Degradation efficiency of diesel vs remediation time during by different bioremediation method. Design I: immobilized bacteria by wood chips; Design II: immobilized bacteria by maize straw; Design III: Free bacteria.

The diesel biodegradation patterns in different remediation methods showed evident differences, as shown in Fig. 3. Different degradation efficiencies of different remediation methods can also be calculated, as shown in Table 2.

**Table 2:** Degradation efficiency percentage (%) and k.

	immobilized bacteria by wood chips	immobilized bacteria by maize straw	Free bacteria
1st stage degradation process	0–15d	0–10d	0–15d
k/d	0.0461	0.0304	0.02848
2ed stage degradation process	15–20d	10–20d	15–20d
k/d	0.00278	0.00295	0.00138

As shown in Fig. 3, the degradation efficiency of diesel followed the order: immobilized bacteria by wood chips (73.39%), immobilized bacteria by maize straw (52.28%) and free bacteria (44.79%). The immobilized bacteria in the wood chips achieved the highest degradation efficiency of diesel (i.e. 73.39%). Obviously, the effect of immobilized bacteria was superior to that of free bacteria. Huang *et al*. reported similar results^19^. The immobilized bacteria in the wood chips performed better than those in the maize straw. In consideration of the surface properties of the carriers and the biomass densities shown in Table 1, a high biomass density contributes to the biodegradation of diesel.

In addition, the degradation in seawater has been divided into a two-stage degradation pattern. As shown in Table 2, the kinetic rates (*k*) were different. The kinetic rates (*k*) were approximated by differentiation of [diesel concentration]/[diesel concentration]_initial_ at each time slot. The *k* of the first stage pattern (0.02848/day–0.0461/day) in each bioremediation method was higher than that of the second stage pattern (0.00138/day–0.00278/day), which indicated that most of the biodegradable or bioavailable compounds were usually rapidly biodegraded during the first stage^20, 21^. In brief, the degradation efficiency of diesel was enhanced by immobilization. By contrast, the high *k* of biodegradation indicated that the bioremediation method of immobilized bacteria in the wood chips was better than the free bacteria method.

In addition, the degradation efficiency constant *k*’s increased slowly in the two-stage pattern, with values of 0.00278/day, 0.00295/day and 0.00138/day. When most of the hydrocarbons were removed in the first stage process, biodegradation occurred slowly.

### Dehydrogenase analysis

Dehydrogenase is an endoenzyme that plays an important role in biodegradation^22^. Generally, organic compounds are dehydrogenated by different types of dehydrogenase. In dehydrogenation, dehydrogenase serves as a catalyst for delivering hydrogen. Thus, dehydrogenase is an important index in the redox system of bacteria.

**Figure 4:**
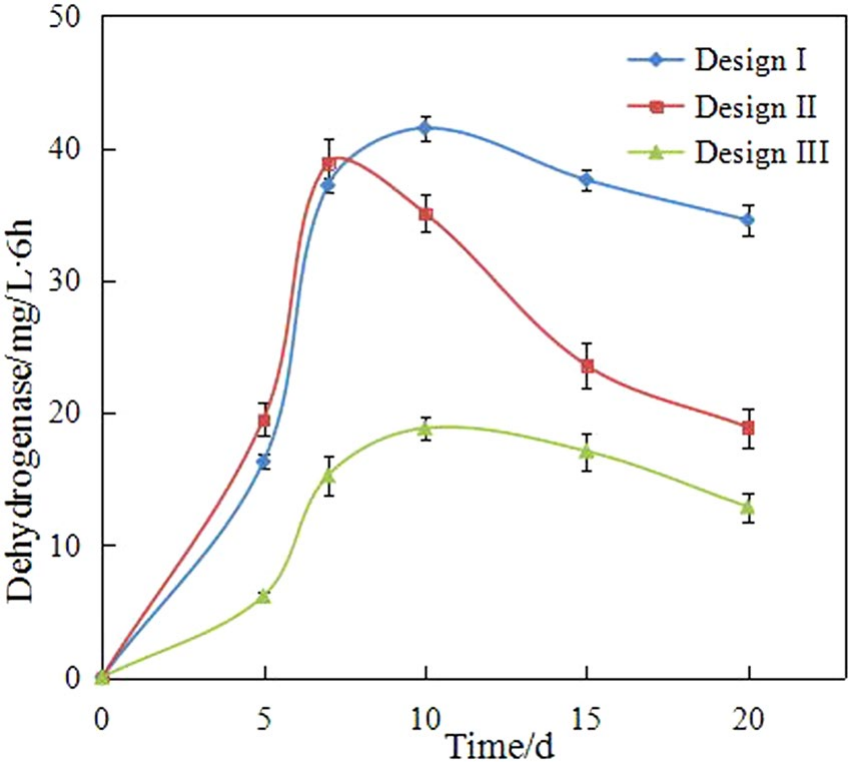
The concentration of dehydrogenase.

As shown in Fig. 4, the concentration of dehydrogenase in immobilization was more than that in the free bacteria system. For example, the concentration of dehydrogenase in the immobilized bacteria ranged from 16.3 mg/(L·6 h) to 41.5 mg/(L·6 h) in the wood chip method but only ranged from 6.15 mg/(L·6 h) to 18.83 mg/(L·6 h) in the free bacteria method. The results showed that the biological activity was higher in immobilization, which could also explain the different degradation efficiencies in the three remediation methods.

In addition, the same change rule was observed in the different remediation methods: dehydrogenase initially increased and then decreased. Taking the immobilized bacteria in the wood chips as an example, the concentration of dehydrogenase initially increased to 37.17 mg/(L·6 h) and then decreased to 34.55 mg/(L·6 h). Although the change rule was the same in immobilization, the concentration of dehydrogenase reached the highest point at 10 days in the wood chips and at 5 days in the maize straw.

### Test for nutrients supplied by carriers

Except for biomass density, nutrient is another important factor used to achieve bioremediation^23^. Lack of nutrients limits the metabolism and affects the activities of bacteria. This study further analysed the importance of nutrients in the immobilization experiments.

**Figure 5:**
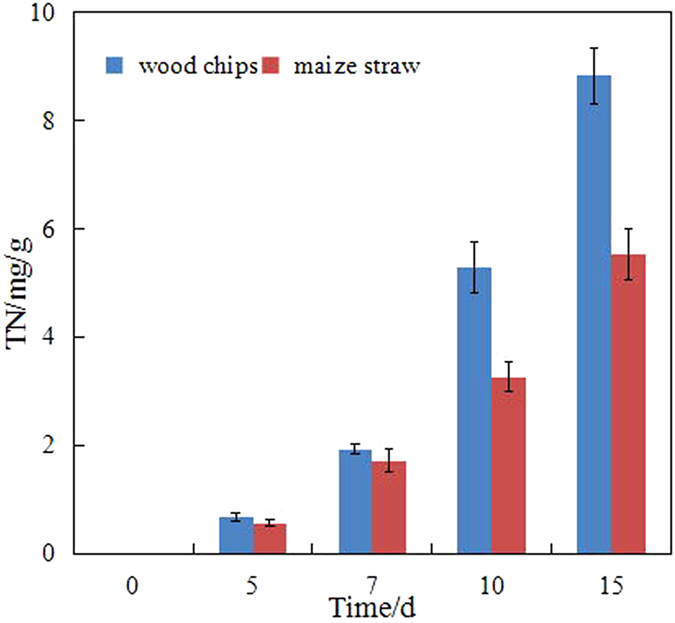
TN released from different carriers.

**Figure 6:**
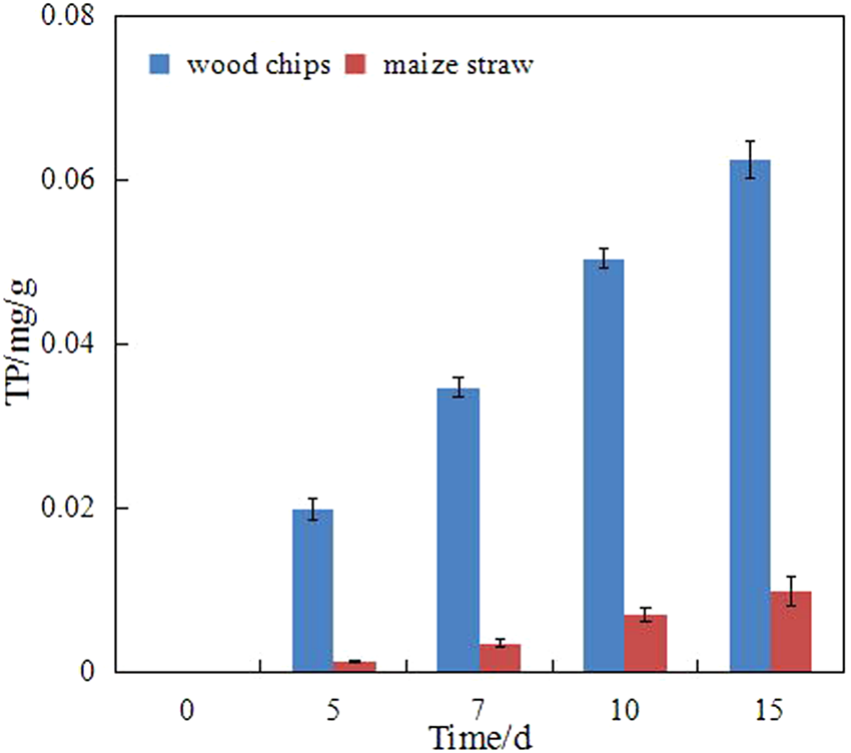
TP released from different carriers.

Firstly, the nutrients released from the carriers (e.g. nitrogen and phosphorus) are presented in Figs 5 and 6. On the basis of the concentrations of TN and TP (Fig. 5), the TN amounts released from the wood chips and maize straw were calculated to be 8.83 and 5.53 mg/g, respectively. Simultaneously, the TP amounts released from the wood chips and maize straw were only 0.0624 and 0.0099 mg/g, respectively. Thus, these carriers could supply some nutrients for diesel-degrading bacteria. However, these nutrients could be under the optimal growth condition (nitrogen/phosphorus = 16:1)^15^.

**Figure 7:**
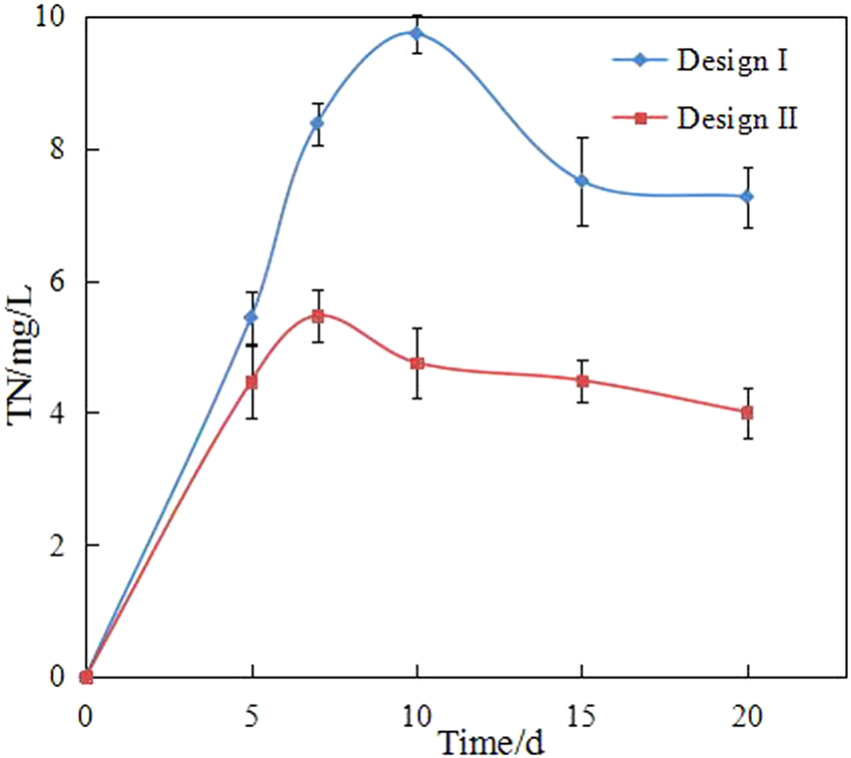
The concentration of TN.

**Figure 8:**
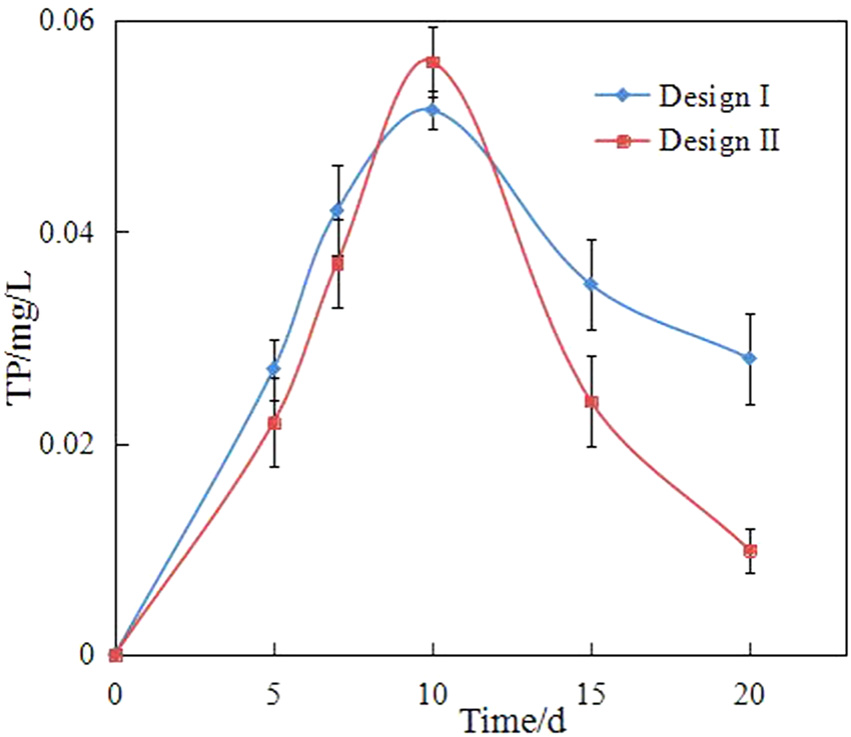
The concentration of TP.

In the biodegradation experiment, we also monitored the concentrations of TN and TP (Figs 7 and 8). In the initial days of the experiment, the concentrations of TN and TP increased from 5.1 mg/L to 9.5 mg/L and from 0.052 mg/L to 0.057 mg/L, respectively. This finding indicated that nitrogen and phosphorus were sufficient. After day 10 or 15, the concentration of TN decreased and showed a tendency toward stabilisation. However, in the final days of the experiment, the concentration of TN was 0.8 times of the highest concentration. By contrast, the concentration of TP was only 0.3–0.4 times of the highest concentration. Considering that the optimal growth condition was nitrogen/phosphorus = 16:1, the concentrations of TN and TP in the end days were low. Therefore, nutrients (particularly phosphorus) should be sufficient to alleviate nutrient limitation during the immobilization and/or bioremediation of oil-polluted seawater and thus enhance biodegradation in future experiments.

## Discussion

Although physical remediation could remove most heavy compounds, the dissolved and suspended hydrocarbons could not be feasibly or efficiently removed from seawater. Bioremediation is an efficient and environmentally friendly remediation technology. However, the marine environment has a certain particularity that limits the bioremediation in oil spill. Firstly, the biomass density in unit space is relatively low that the activity and effect of biodegradation are affected by the influence of hydrologic conditions (e.g. winds and currents) and the quality of seawater (e.g. salinity). Secondly, nutrient is an important limiting factor, which is usually low in seawater. Immobilization was introduced and intensively investigated as an alternative approach that could enhance the biomass density and supply nutrients during biodegradation. The biomass densities of the wood chips and maize straw were 0.0242 and 0.0097 g VSS/g carrier, respectively. The amounts of TN released from the wood chips and maize straw were 8.83 and 5.53 mg/g, respectively. Simultaneously, the amounts of TP released from the wood chips and maize straw were only 0.0624 and 0.0099 mg/g, respectively. Thus, the diesel-degrading bacteria immobilized on the natural organic carriers were investigated. The results indicated that the degradation efficiency of diesel by immobilization was apparently higher than that by free bacteria. This result can be ascribed to two reasons:

Firstly, the suitable distributions of porosity in natural organic carriers are beneficial to adsorb bacteria. The biofilm formed on the carrier surface could enhance biomass density. In addition, diesel could be dispersed and adsorbed on the surface of the carriers because of their small diameter and high specific surface area. Many face-to-face opportunities between bacteria and diesel are provided in unit space. Notably, biomass density and biodegradation efficiency vary with the different surface properties of different carriers (e.g. specific surface area and median diameter of the adsorption pore). Several researchers reported that surface properties influence biodegradation efficiency^24^. Therefore, the effect of surface properties should be intensively investigated.

Secondly, nutrient released from natural organic carriers improves diesel-degrading bacterial activity. Natural organic carriers, such as wood chips and maize straw, are excellent sources of proteins and fibrin. In contrast to inorganic carriers, several nutrients that could be utilised by diesel-degrading bacteria are released from these natural organic carriers. Thus, the dehydrogenase activity and biodegradation efficiency are high in immobilization. In the present study, the degradation efficiencies of diesel were in the order of immobilized bacteria by wood chips (73.39%), immobilized bacteria by maize straw (52.28%) and free bacteria (44.79%). However, the amounts of nutrient (e.g. nitrogen and phosphorus) were not the optimal biodegradation condition. In particular, the metabolism of bacteria during the stabilisation period (after 10 days) was limited by the lack of sufficient nutrients, which decreased diesel biodegradation efficiency. Natural organic carriers should be modified to improve biodegradation further. For example, nitrogen and phosphorus should be added to release the nutrients of natural organic carriers for diesel-degrading bacteria.

In brief, immobilization by natural organic carriers can be used to remove diesel in the marine environment. The technology not only allows bacteria to be adsorbed and form biofilm on the carrier surface but also provides nutrients for the bacteria, which promote bioremediation in the marine environment.

## Conclusions

This study investigated the diesel-degrading bacteria immobilized on natural organic carriers (i.e. wood chips and maize straw) to improve the efficiency of immobilization in the marine environment. The characteristics of diesel-degrading bacteria immobilized on natural organic carriers were assessed. The degradation activity of the bacteria was analysed, and the effect of the carriers was discussed. The conclusions are as follows:

1. The surface properties of the wood chips and maize straw showed that the carriers, i.e. wood chips and maize straw, are suitable for immobilization technology. The biomass densities of the wood chips and maize straw were 0.0242 and 0.0097 g VSS/g carrier, respectively.
2. The biodegradation efficiency of immobilization was higher than that of free bacteria. The degradation efficiencies of diesel were in the order of immobilized bacteria by wood chips (73.39%), immobilized bacteria by maize straw (52.28%) and free bacteria (44.79%). The degradation in seawater was divided into a two-stage degradation pattern. *k* of the first stage pattern (0.02848/day–0.0461/day) in each bioremediation method was higher that of the second stage pattern (0.00138/day–0.00278/day), which indicated that most of the biodegradable or bioavailable compounds were usually rapidly biodegraded during the first stage.
3. The biological activity in immobilization was high, which could also explain the high degradation efficiency. The concentration of dehydrogenase in immobilization was more than that in the free bacteria system.
4. By tracing its cause, this study determined that the amounts of TN released from wood chips and maize straw were 8.83 and 5.53 mg/g, respectively, and those of TP released from wood chips and maize straw were 0.0624 and 0.0099 mg/g, respectively. The results showed that natural organic carriers can provide nutrients for bacteria.
